# Supplementary material for: The centriculum, a membrane reticulum that surrounds Caenorhabditis elegans centrosomes, might serve as a microtubule filter
Source: J Cell Sci. 2026 Jul 10;139(13):jcs264404. doi: 10.1242/jcs.264404 (PMC13380966; doi:10.1242/jcs.264404)
Supplement: Supplementary information [file joces-139-264404-s1.pdf]

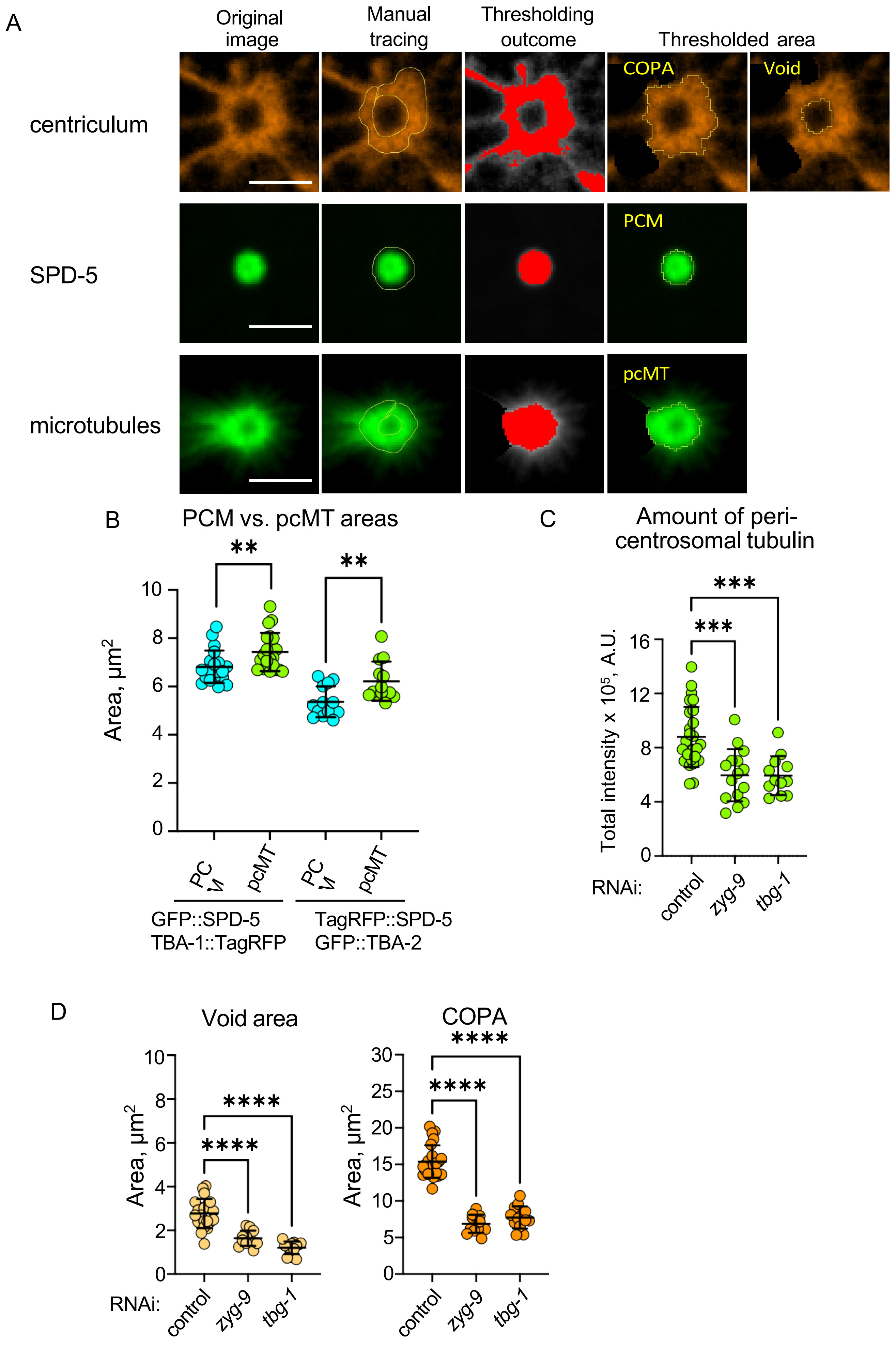
A
Original image
Manual tracing
Thresholding outcome
Thresholded area
centriculum
COPA
Void
SPD-5
PCM
microtubules
pcMT
B
PCM vs. pcMT areas
Area, μm2
PCM
pcMT
GFP::SPD-5
TBA-1::TagRFP
TagRFP::SPD-5
GFP::TBA-2
C
Amount of peri-centrosomal tubulin
Total intensity x 10^5, A.U.
RNAi:
control
zyg-9
tbg-1
D
Void area
COPA
Area, μm2

Fig. S1. Areas of the PCM, pcMT, centriculum void area and COPA under control, *zyg-9* and *tbg-1* RNAi conditions.

A) Images of a centriculum, PCM and microtubules of a 1-cell stage metaphase embryos demonstrating how manual tracing and thresholding is used to measure these structures' areas. See the Material and Methods section for the detailed process. Scale bar=5 μm.

B) Comparison of PCM and pcMT areas using different fluorescent proteins pairs: OCF247: GFP::SPD-5; TBA-1::tagRFP (same data as shown in Fig 1E, n=20, p=0.0043); and OCF240: RFP::SPD-5; GFP::TBA-2) n=13, p=0.0089, using the Mann-Whitney test.

C) Quantification of the amount of peri-centrosomal tubulin fluorescence for the images such as shown in Fig 2A, using strain OCF181. n= 26, 15, and 12 for control, zyg-9 and tbg-1 RNAi treatments, respectively. p=0.0001 for control vs. zyg-9 and p=0.0003 for control vs. tbg-1 RNAi treatments using ordinary one-way ANOVA. Error bars represent mean and standard deviation.

D) Quantification of the void area and COPA for the images such as shown in Fig 2D, using strain OCF176. For void area: n= 22,12, and 14 for control, *zyg-9* and *tbg-1* RNAi treatments, respectively. p<0.0001 for control vs. *zyg-9* and for control vs. *tbg-1* RNAi treatments using ordinary one-way ANOVA. For COPA: n= 23,12, and 14 for control, *zyg-9* and *tbg-1* RNAi treatments, respectively. p<0.0001 for control vs *zyg-9* and for control vs *tbg-1* RNAi treatments using ordinary one-way ANOVA. Error bars represent mean and standard deviation. Note that the data for the control RNAi are the same as shown in Fig 1D.

Journal of Cell Science • Supplementary information

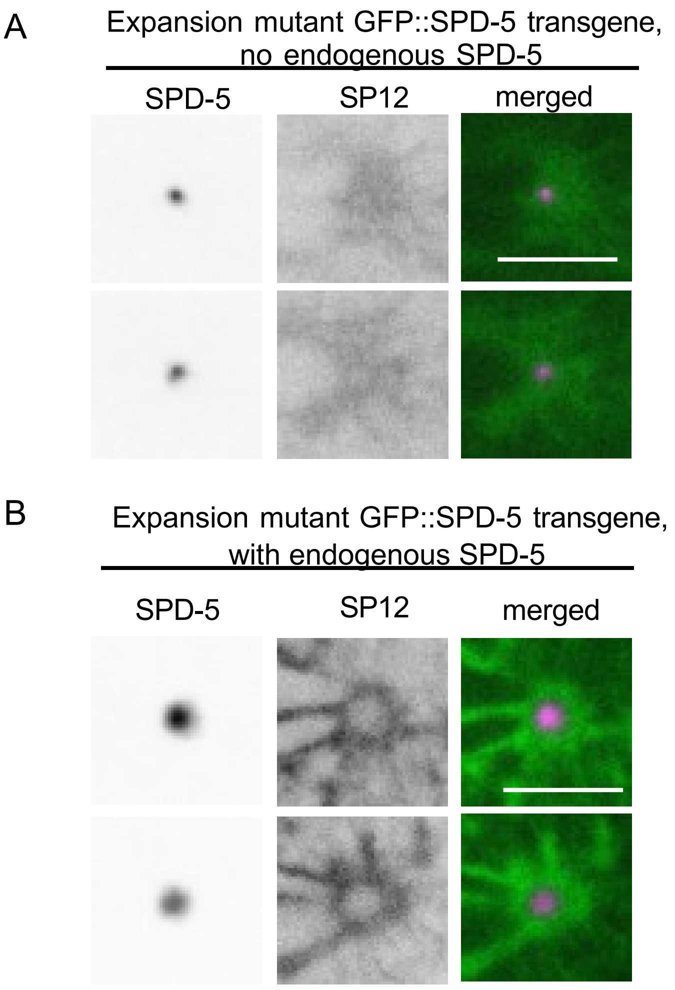

Fig. S2. The effect of endogenous SPD-5 on the distribution of transgenic expansion mutant SPD-5.

A and B) Additional examples of centricula and PCM from 1-cell embryos at metaphase expressing mCherry::SP12 (green in merged images) and transgenic GFP::SPD-5^{exp} (magenta in merged images) without (panel A) or with (panel B) endogenously expressed SPD-5 (OCF 189), as also shown in Fig 3C and D. Endogenous spd-5 was down-regulated in panel A using RNAi. Scale bar=5 μm.

Journal of Cell Science • Supplementary information

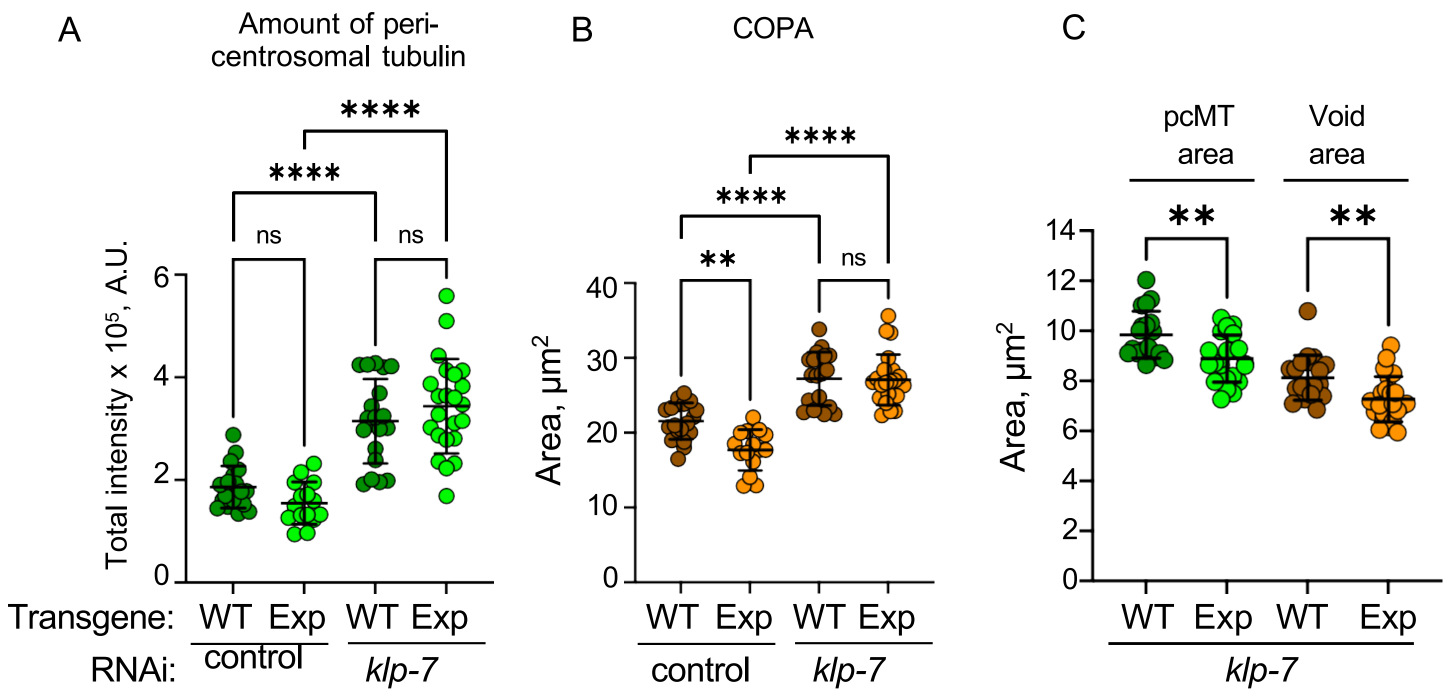

Fig. S3. pcMT, COPA and centriculum void area in embryos from control and klp-7 *RNAi* treated worms.

A and B) Quantification of peri-centrosomal tubulin amount and COPA from the control and *klp-7* RNAi treated embryos as shown in Fig 5B (strains OCF214 and 215). n=19 and 15 for wild type and *spd-5*^{exp} transgenes, respectively, treated with control RNAi. n=20 and 23 wild type and *spd-5*^{exp} transgenes, respectively, treated with *klp-7* RNAi. p-values, determined by ordinary one-way ANOVA, are (from left to right): panel A: 0.5932, <0.0001, <0.0001 and 0.5529; panel B: 0.0012, <0.0001, <0.0001 and 0.8739. Error bars represent mean and standard deviation.

C) Quantification of pcMT area and void area following *klp-7* RNAi treatment as shown in Fig 5B. n=20 and 23 for wild type and *spd-5*^{exp} transgenes, respectively. p-values are 0.0021 (pcMT area) and 0.0061(void area) as determined by ordinary one-way ANOVA. Error bars represent mean and standard deviation

Journal of Cell Science • Supplementary information

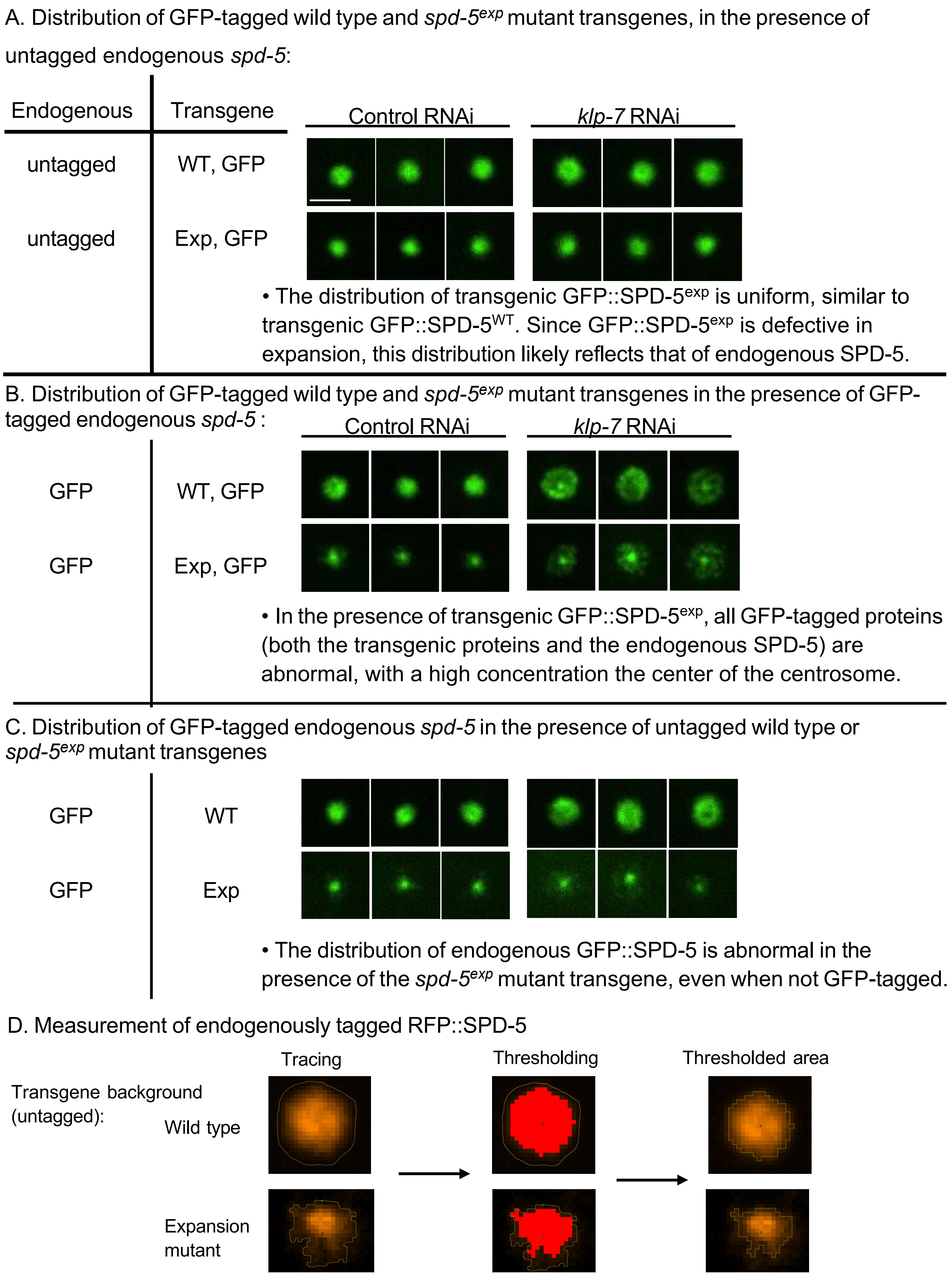

Journal of Cell Science • Supplementary information

Fig. S4. The effect of SPD-5exp on endogenous SPD-5::GFP.

A-C) A comparison of endogenous and/or transgenic *spd-5* distribution when fused to GFP, in the presence of untagged or wild type or *spd-5*^{exp} transgenes. Images are of three different centrosomes following control or *klp-7* RNAi treatment. The latter allows for more detailed protein distribution. The strains used were as follows: panel A: OCF187 and OCF189, expressing the wild type or *spd-5*^{exp} transgene, respectively, both tagged with GFP and expressing untagged endogenous *spd-5*; panel B: OCF221 and OCF223, expressing the wild type or *spd-5*^{exp} transgene, respectively, both tagged with GFP and expressing GFP-tagged endogenous *spd-5*; and panel C: OCF218 and OCF227, expressing the untagged wild type or *spd-5*^{exp} transgene, respectively, and GFP-tagged endogenous *spd-5*. Scale bar=5 µm.

D) Steps to quantify PCM area of centricula of 1-cell embryos expressing RFP::SPD-5 with either transgenic wild type *spd-5* (OCF234) or untagged *spd-5*^{exp} mutant (OCF233). See Materials and Methods for detailed quantification process.

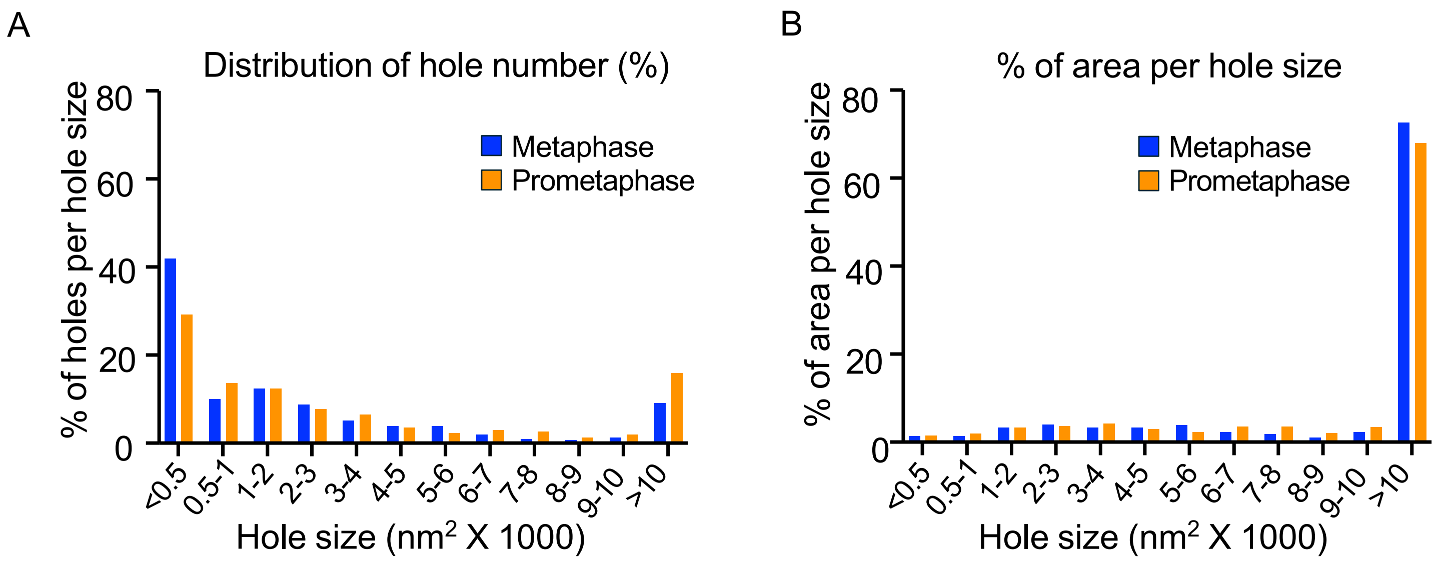

Fig. S5. Hole size distribution in prometaphase and metaphase centricula.

A) Binned frequency distribution of holes present on the pronuclear side of metaphase (in blue) and prometaphase (orange) centricula from 1-cell embryos. The metaphase data are the same as shown in Fig 7D. Bin size range is shown on the x-axis. n=308 for prometaphase holes; pooled from 6 images taken from 3 centricula (2 images per centriculum).

B) Binned frequency distribution of the percentage of total open area per hole size range, for holes in metaphase (in blue) and prometaphase (orange) centricula from 1-cell embryos, using the same data as in panel A. The data for metaphase centricula are the same as shown in Fig 7E.

Table S1. ***C. elegans strains***

***C. elegans* strains**		
**Name**	**Genotype**	**Source**
N2	wild type (Bristol)	CGC
MSN146	ltIs76 [pAA178: *pie-1p::mCherry::SP12 + unc-119* (+)]; ltIs25 [pAZ132; *pie-1p::GFP::tba-2 + unc-119 (+)*];*unc-119(ed3)*III	Jon Audhya lab
OCF176	*spd-5(vie26[GFP::spd-5 +loxP]) I; ocfIs2[pie-1p::mCherry::SP12::pie-1 3'UTR +unc119 (+)];his-72(erb77[his-72::linker::mTurquoise2]III; unc-119(ed3)III*	(Maheshwari *et al.*, 2023)
OCF181	*ocfIs2[pie-1p::mCherry::SP12::pie-1 3'UTR +unc119 (+)]; ltIs25 [pAZ132; pie-1p::GFP::tba-2 + unc-119 (+)];his-72(erb77[his-72::linker::mTurquoise2]III; unc-119(ed3)III*	(Maheshwari *et al.*, 2023)
OCF183	*ocfIs2[pie-1p::mCherry::SP12::pie-1 3'UTR +unc119 (+)];ltIs25 [pAZ132; pie-1p::GFP::tba-2 + unc-119 (+)]; his-72(erb77[his-72::linker::mTurquoise2]III; ocf101[atln-1::3xFLAG::degron] IV (CRISPR); TIR1::mRuby IV; unc-119(ed3)III*	(Maheshwari *et al.*, 2023)
OCF187	*ocfIs2[pie-1p::mCherry::SP12::pie-1 3'UTR +unc119 (+)]; cb-unc-119(+)]I; ltSi1141[pOD1021/pVV103; spd-2p::GFP::spd-5 reencoded; cb-unc-119(+)]II; unc-119(ed3)III*	This study
OCF189	*ocfIs2[pie-1p::mCherry::SP12::pie-1 3'UTR +unc119 (+)]; ltSi592[pVV168; spd-2p::GFP-spd-5 mut 653,658, reencoded; cb-unc-119(+)]II; unc-119(ed3)III*	This study
OCF193	*ojIs23 [pie-1p::GFP::SP12 + unc-119(+)]; GFP::PH; tba-1(pg77[tba-1::TagRFP-T + loxP]) I; unc-119(ed3)III*	This study
OCF200	*ltSi592[pVV168; spd-2p::GFP::spd-5 mut 653,658, reencoded; cb-unc-119(+)]II; spd-5(wow36[tagRFP-T^3xmyc::spd-5])I; unc-119(ed3)III;*	This study
OCF201	*cb-unc-119(+)]I; ltSi1141[pOD1021/pVV103; spd-2p::GFP::spd-5 reencoded; cb-unc-119(+)]II; spd-5(wow36[tagRFP-T^3xmyc::spd-5])I; unc-119(ed3)III*	This study
OCF212	*tba-1(pg77[tba-1::TagRFP-T + loxP]) I; spd-2p::GFP::spd-5 reencoded; cb-unc-*	This study

Journal of Cell Science • Supplementary information

	119(+)]II; unc-119(ed3) III	
OCF213	*tba-1(pg77[tba-1::TagRFP-T + loxP]) I; ltSi592[pVV168; spd-2p::GFP::spd-5 mut 653,658, reencoded; cb-unc-119(+)]II; unc-119(ed3)III*	This study
OCF214	*ojIs23 [pie-1p::GFP::SP12 + unc-119(+)];tba-1(pg77[tba-1::TagRFP-T + loxP]) I; ltSi1129[pZZ2; spd-2p::spd-5 (re-encoded);cb-unc-119(+)]II; unc-119(ed3)III*	This study
OCF215	*ojIs23 [pie-1p::GFP::SP12 + unc-119(+)]; tba-1(pg77[tba-1::TagRFP-T + loxP]) I; ltSi1219[pMO104; spd-2p::spd-5 S653A S658A::spd-5 3'UTR; cb-unc119(+)]II; unc-119(ed3)III*	This study
OCF218	*ltSi1129[pZZ2; spd-2p::spd-5 (re-encoded);cb-unc-119(+)]II; spd-5(vie26[GFP::spd-5 +loxP]) I; ocfIs2[pie-1p::mCherry::SP12::pie-1 3'UTR +unc119 (+)]; unc-119(ed3)III*	This study
OCF221	*cb-unc-119(+)]I; ltSi1141[pOD1021/pVV103; spd-2p::GFP::spd-5 reencoded; cb-unc-119(+)]II; spd-5(vie26[GFP::spd-5 +loxP]) I; ocfIs2[pie-1p::mCherry::SP12::pie-1 3'UTR +unc119 (+)];unc-119(ed3)III*	This study
OCF223	*ltSi592[pVV168; spd-2p::GFP::spd-5 mut 653,658, reencoded; cb-unc-119(+)]II; spd-5(vie26[GFP::spd-5 +loxP]) I; ocfIs2[pie-1p::mCherry::SP12::pie-1 3'UTR +unc119 (+)];unc-119(ed3)III*	This study
OCF227	*ltSi1219[pMO104; spd-2p::spd-5 S653A S658A::spd-5 3'UTR;cb-unc-119(+)]II; spd-5(vie26[GFP::spd-5 +loxP]) I; ocfIs2[pie-1p::mCherry::SP12::pie-1 3' UTR +unc119 (+)],unc-119(ed3)III*	This study
OCF233	*ojIs23 [pie-1p::GFP::SP12 + unc-119(+)]; ltSi1219[pMO104; spd-2p::spd-5 S653A S658A::spd-5 3'UTR;cb-unc-119(+)]II; spd-5(wow36[tagRFP-T^3xmyc::spd-5])I; unc-119(ed3)III*	This study
OCF234	*ojIs23 [pie-1p::GFP::SP12 + unc-119(+)]; ltSi1129[pZZ2; spd-2p::spd-5 (re-encoded);cb-unc-119(+)]II; spd-5(wow36[tagRFP-T^3xmyc::spd-5])I; unc-119(ed3)III*	This study
OCF240	*spd-5(wow36[tagRFP-t^3xmyc::spd-5]) I; ltIs25 [pAZ132; pie-1p::GFP::tba-2 + unc-119 (+)]*	This study
OCF247	*spd-5(wow52[GFP^3xflag::spd-5]) I tba-1(pg77[tba-1::TagRFP-T + loxP]) I*	This study
